# Correlations among metamorphopsia test scores, optical coherence tomography findings and multifocal electroretinogram responses in epiretinal membrane patients

**DOI:** 10.1007/s10633-020-09803-x

**Published:** 2021-01-03

**Authors:** Jung Woo Lee, Sung Yong Park, Patrick S. Kim, In Hwan Cho, Hoon Dong Kim

**Affiliations:** 1 Department of Ophthalmology, Soonchunhyang University College of Medicine, Cheonan Hospital, 31, Suncheonhyang 6-gil, Dongnam-gu, Cheonan-si, Chungcheongnam-do, Cheonan, 31151 South Korea; 2 Yesungjung Eye Clinic, Seoul, South Korea; 3 Department of Ophthalmology, Chuncheon Sacred Heart Hospital, Hallym University College of Medicine, Chuncheon, South Korea

**Keywords:** Epiretinal membrane, Metamorphopsia, Multifocal electroretinogram, Central retinal thickness

## Abstract

**Purpose:**

To quantify metamorphopsia with a novel objective method in patients with epiretinal membrane (ERM) and to compare the relationships among metamorphopsia scores, spectral-domain optical coherence tomography (OCT) findings, and multifocal electroretinogram (mfERG) results.

**Methods:**

This study included 52 eyes of 52 patients with idiopathic ERM who underwent comprehensive ophthalmologic examinations, including measurement of best-corrected visual acuity (BCVA), OCT, and mfERG. The degree of metamorphopsia was quantified using MonPack One® (Metrovision, Perenchies, France). On the topographic map of the early treatment diabetic retinopathy (ETDRS) grid, retinal thickness in the central, superior, inferior, nasal, and temporal subfields were measured, and metamorphopsia scores for each corresponding subfield were also obtained. The amplitudes and implicit times of mERG were elicited from each subfield. Then, the correlations among metamorphopsia scores, OCT findings, and mfERG responses were analyzed.

**Results:**

The mean age of the patients was 65.3 ± 18.5 y, and the average metamorphopsia score of the individual subfields was 2.03 ± 1.18. Initial BCVA was 0.50 ± 0.12 logMAR, but there was no significant correlation between metamorphopsia scores and BCVA. The metamorphopsia scores from the central subfields showed significant correlations with central retinal thickness (CRT) (*p* = 0.001). The mean metamorphopsia scores in the central subfield showed a significant relationship with the mean N1 and P1 amplitudes (*p* = 0.001, *p* = 0.048, respectively), while no relationship was observed between metamorphopsia scores and mfERG amplitudes in other subfields.

**Conclusions:**

The degree of metamorphopsia in patients with ERM could be objectively quantified in each subfield using a novel metamorphopsia test. The metamorphopsia scores were significantly correlated with retinal thickness, especially at the central subfields, and the scores in the central subfields were significantly correlated with the N1 and P1 amplitudes of mfERG. Thus, the metamorphopsia test can be a useful method to evaluate metamorphopsia symptoms for patients with ERM.

## Introduction

Epiretinal membrane (ERM) is a pathologic condition characterized by avascular and fibrocellular membranes that proliferate along the surface of the internal limiting membrane (ILM) of the retina. It causes retinal wrinkle, traction, and distortion of the fovea and can induce symptoms such as decreased vision, monocular diplopia, and metamorphopsia [1]. Metamorphopsia is one of the most common symptoms in patients with ERM, and about 80–85% of the patients complain of moderate to severe bends [2, 3]. In many patients, even when visual acuity improves after successful surgical removal of the ERM, metamorphopsia symptoms may persist.

Quantification of metamorphopsia symptoms is essential for estimating the visual function, determining the disease progression, and assessing the treatment outcomes in the patients with ERM. Several methods have been proposed to objectively evaluate metamorphopsia in the patients. The Amsler grid, which has been mainly used in these assessments, offers the advantage of being intuitive and very simple to perform [2]. However, it is a subjective test and has shown low sensitivity and limitations in quantifying the severity of metamorphopsia. Preferential hyperacuity perimetry (PHP) is a psychophysical test that uses visual hyperacuity to identify and quantify visual disturbances such as metamorphopsia and scotoma [4]. In this test, the severity of metamorphopsia in the area of interest can be quantified by manipulating the amplitude of the screen. However, PHP is difficult to perform for elderly patients and requires expensive equipment. The M-chart has also been used as a clinical tool for quantification in patients with macular diseases. However, the M-chart shows limited objective quantification, and metamorphopsia tests using the M-chart can be performed only in patients with visual acuity better than 20/100 without large scotomas [5]. Recently, a novel objective method for metamorphopsia testing installed in electrophysiology machine (MonPack One®, Metrovision, Perenchies, France) was introduced. Using this method, metamorphopsia scores are obtained for multiple regional areas in the central retina after the test.

Optical coherence tomography (OCT) is a useful modality for diagnosing and assessing the progression of macular diseases including ERM, and it can evaluate the retinal structures including retinal thickness, retinal volume, and the integrity of the photoreceptor cell layer. With the development of spectral domain OCT (SD-OCT), higher-resolution examinations within less time have become possible, encouraging research on the microstructure of the retina and visual function [6–8].

In addition, multifocal electroretinogram (mfERG) allows noninvasive and objective detection of regional retinal dysfunction, especially in the central retina [9]. In a previous study, delay in the P1 implicit time from mfERG was a significant predictor of poor visual recovery after ERM surgery [10]. mfERG can be used to investigate the pathophysiology of ERM and to evaluate the degree of functional decline in the macula on SD-OCT [11].

The purpose of this study was to objectively quantify the degree of metamorphopsia symptoms using the novel metamorphopsia testing method in patients with ERM. Furthermore, the correlations among metamorphopsia scores from each retinal region, SD-OCT findings obtained with quantitative sequencing, and mfERG parameters were also investigated in this study.

## Methods

This cross-sectional observational study was performed between July 2018 and June 2020 and enrolled patients who had received a clinical diagnosis of unilateral idiopathic ERM by means of ophthalmoscopy and OCT. Patients with secondary ERM caused by other retinal etiologies, previous history of vitreoretinal surgery, and other comorbid macular diseases, including retinal detachment, retinal vascular diseases, diabetic retinopathy, macular hole, and age-related macular degeneration were excluded from this study. Patients who had nuclear sclerosis of grade 2 or higher on Lens Opacities Classification System (LOCS) III, glaucoma, and refractive error greater than 6 diopters were also excluded. To properly perform the metamorphopsia test, patients with best-corrected visual acuity (BCVA) lower than 20/200 were excluded from this study. The normal fellow eyes without ocular morbidity served as controls. The study was approved by the institutional review board (IRB) (IRB No. 2020–07-045) and adhered to the tenets of the Declaration of Helsinki. Informed consent was obtained from all individual patients included in this study at enrollment.

### Ophthalmic examinations

All patients who met the abovementioned criteria underwent comprehensive ophthalmic examinations, including BCVA, intraocular pressure (IOP) measurement using noncontact tonometry, slit-lamp biomicroscopy, and ophthalmoscopy. BCVA was measured using the Early Treatment Diabetic Retinopathy Study (ETDRS) charts at a 4 m distance. The results of visual acuity examinations were converted into logarithm of the minimum angle of resolution (LogMAR) units for statistical analysis.

### Metamorphopsia test

**Fig. 1:**
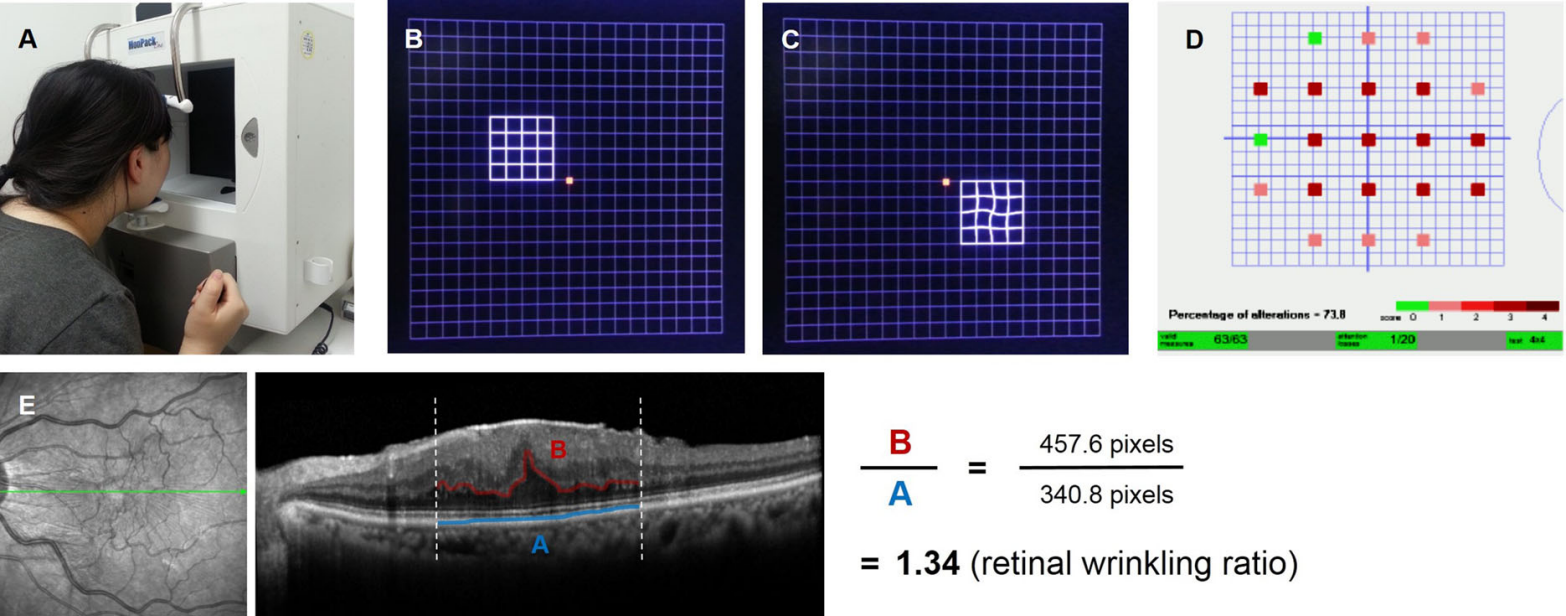
Methodology of the metamorphopsia test and calculation of the retinal wrinkling ratio on OCT horizontal scans. The metamorphopsia test, a novel method used for quantification of metamorphopsia, is similar to automatic visual field perimetry (**A**). White stimulation consisting of a 4 × 4 grid pattern of straight or curved lines under a dark blue background was displayed at each subfield on the monitor with a pseudo-random sequence (**B**, **C**). The patient holds the response button while keeping the eyes on the central red-colored gaze point and presses the button when the grid pattern with curved lines is recognized. Metamorphopsia scores for 21 subfields were presented as 2 points for green, 1 point for pink, and 0 points for red (**D**). The retinal wrinkling ratio was calculated based on the lengths of the inner boundary of the outer nuclear layer (ONL) and the length of the RPE layer measured on the OCT scan. The length of the inner boundary of the ONL was divided by that of the RPE layer (**E**)

The metamorphopsia test installed in MonPack One® machine uses a novel method for quantification of metamorphopsia, similar to automatic visual field perimetry examination (Fig. 1a). The overall testing field is divided into 21 subfields under a dark blue background. White stimulation consisting of a 4 × 4 grid pattern of straight or curved lines is displayed at each subfield on the monitor with a pseudo-random sequence (Fig. 1b, c). The patient holds the response button while keeping the eyes on the central red-colored gaze point and presses the button when the grid pattern with straight lines is recognized. The outermost line of each grid represents a range of 10° in each direction. Each line spacing shows a difference of 1°, and each inspection point is 4° away from the next point. Three tests are performed at each subfield. A curved pattern is presented once to confirm the reliability of the test, and straight linear patterns are presented twice to detect the patient’s metamorphopsia. A green point is shown if the patient responds twice for the straight linear pattern, a pink point represents a single response for the straight linear pattern, and a red point means that there was no response for the straight linear patterns. Metamorphopsia scores for the 21 subfields were scored as 2 points for green, 1 point for pink, and 0 points for red. The patients were tested twice for the straight linear pattern measures; thus, metamorphopsia scores ranged from 0 to 4 for the 21 subfields on the result paper (Fig. 1d). The number of tests presented in a curved pattern is expressed as “attention losses,” and attention losses are recorded even for cases involving abnormally fast reactions. Therefore, higher metamorphopsia test scores may indicate more severe metamorphopsia symptoms, and lower scores may represent mild symptoms in the patients with ERM. The relationships between the mean retinal thickness using OCT scans in the superior, inferior, nasal, and temporal parafoveal areas and metamorphopsia scores at each corresponding subfield were evaluated.

In addition, the patients were divided into subgroups based on the severity of metamorphopsia in the central subfield, namely the no-metamorphopsia group (metamorphopsia score, 0), mild-metamorphopsia group (score, 1–2), and severe-metamorphopsia group (score, 3–4). The alterations in OCT findings and mfERG results were compared according to the subgroups for metamorphopsia severity.

### Optical coherence tomography

SD-OCT (Spectralis® OCT, Heidelberg Engineering Inc., Heidelberg, Germany) was performed with a raster scan consisting of 31 B-scans in the 30° × 25° zone centered on the fovea. Each B-scan consisted of 768 A-scans of 9 mm length, and each scan interval was 240 μm. Through the automatic real time (ART) mode using an eye tracker system, 25 frames were averaged to create one B-scan. The test was performed by a single skilled technician who was blinded to patient information, and the thickness of the retina was measured using installed software. The central (foveal) area of the ETDRS grid and the parafoveal area within the grid were measured. Central retinal thickness (CRT, μm) was obtained from the cube scan results, and the mean retinal thickness values of the superior, inferior, nasal, and temporal grids were also estimated. Additionally, the retinal wrinkling ratio was calculated based on the findings of the OCT horizontal raster scan images across the macula (Fig. 1e). The lengths of the inner boundary on the outer nuclear layer (ONL) and the retinal pigment epithelium (RPE) layers were measured manually by a single reader using ImageJ (National Institutes of Health, Bethesda, MD). Subsequently, the length of the inner boundary on the ONL was divided by that of the RPE layer (Fig. 1e). The correlation of the retinal thickness and retinal wrinkling ratio on OCT scan images with the metamorphopsia scores and mfERG parameters in patients with ERM were investigated.

### Multifocal electroretinogram

The first-order kernel mfERG responses were elicited using MonPack One®, according to the standard document of the International Society for Clinical Electrophysiology of Vision (ISCEV) for mfERG recording [9]. Prior the mfERG recording, the eyes were light-adapted for at least 15 min in room light, and the pupils of the patients were fully dilated with 1% tropicamide and 2.5% phenylephrine hydrochloride. Refractive correction was provided at the time of the test. Corrective lenses were placed in a holder positioned in front of the eye with proper centration. The recording was performed by an experienced investigator using contact lens electrodes (ERG jet®, Fabrinal SA, La Chaux-de-Fonds, Switzerland) after anesthetizing the cornea with proparacaine 0.5% eye drops.

The stimulus consisted of an array of 61-scaled hexagon-based patterns presented on a liquid crystal display (LCD) monitor with a frame frequency of 75 Hz. The luminance of the stimulus for white was 200 cd/m^2^, and the contrast was 99.3%. A 61-scaled hexagonal stimulus with a central fixation point at a viewing distance of 33 cm (corresponding to a field of ± 30° horizontally and ± 24° vertically) was used. The luminance of the bright hexagon was maintained at 100 cd/m^2^ while that for the dark hexagon was < 1 cd/m^2^ and that for the background cover was 30 cd/m^2^. The stimulus frequency was set at 17 Hz. The bandpass of the filters was 3 to 100 Hz, and amplification was performed with a gain of 105. Fixation stability was continuously monitored with a mounted infrared camera during the recording.

The mfERG results, including the amplitude and implicit times of the P1 and N1 responses, were evaluated. The N1 amplitude was measured from the baseline to the N1 trough, and the P1 amplitude was measured from the N1 trough to the P1 peak, and they were expressed as response density per unit area (nV/deg^2^). The implicit times of the N1 and P1 waves were presented by measuring the time from the onset of the stimuli and expressed in milliseconds (ms). Averaged values were used after five repeated recordings to increase the reliability of the analyses.

**Fig. 2:**
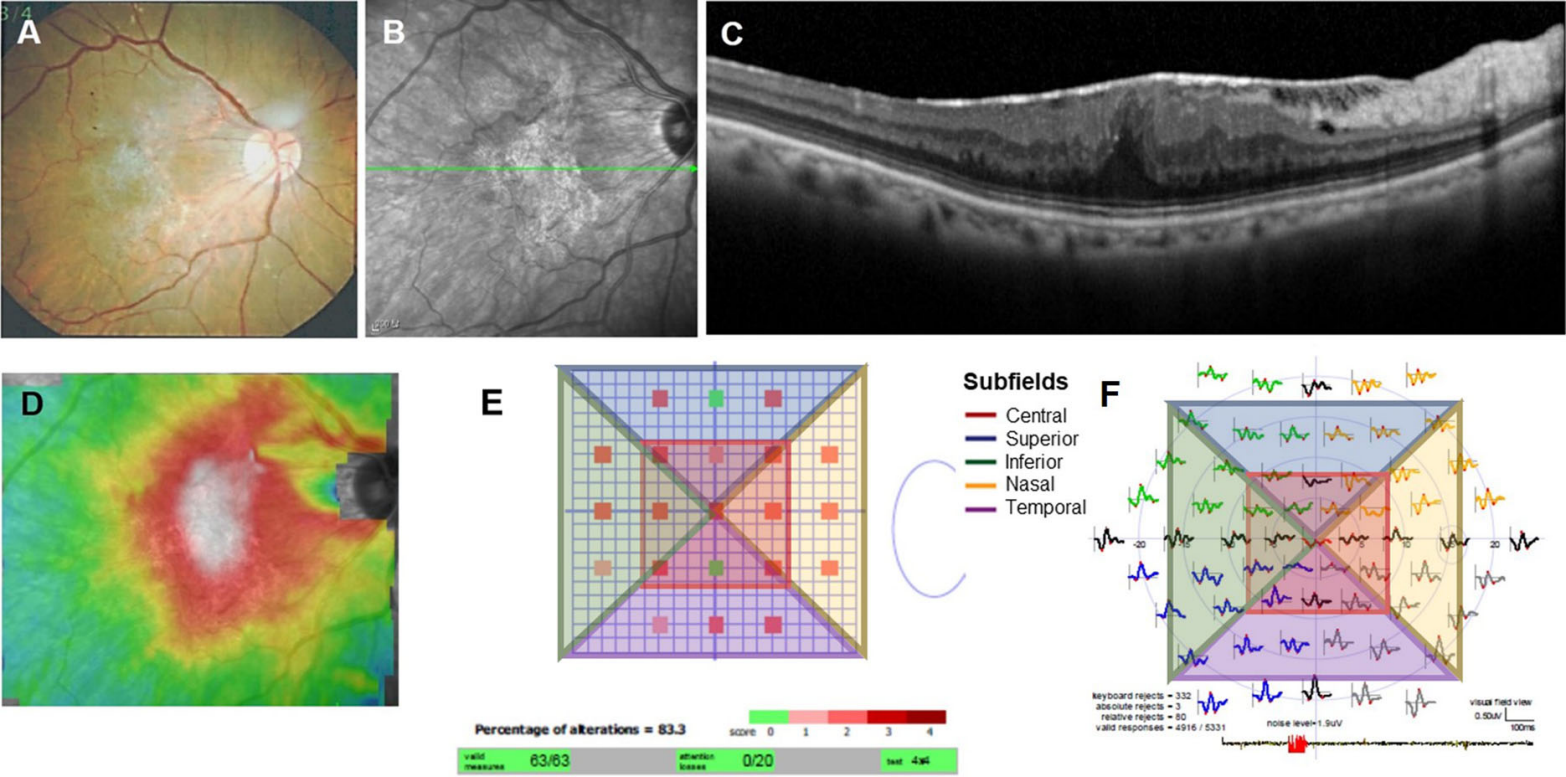
Representative clinical findings for a 62-year-old patient with idiopathic ERM. A thickened fibrovascular membrane was found on fundus examination (**A**) and OCT scan (**B**, **C**) with a deviation map (**D**). Metamorphopsia scores for 21 subfields were obtained (**E**), and mfERG responses were also elicited from the patient (**F**). The results of the metamorphopsia test and mERG recordings were analyzed after dividing the test field of the retina into central, superior, inferior, nasal, and temporal subfields (**E**, **F**)

Individual mfERG responses for the hexagons were grouped into five concentric rings centered on the fovea for analysis (ring 1 representing the < 2° field, ring 2 representing the 2°–5° field, ring 3 representing the 5°–10° field, ring 4 representing the 10°–15° field, and ring 5 representing the > 15° field). In each concentric ring, responses from the foveal (ring 1) and parafoveal (ring 2) areas that mainly reflect the macular function were obtained. In this study, averaged amplitudes and implicit times from ring 1 and ring 2 were compared with the central metamorphopsia score and CRT. After dividing the testing field into the central, superior, inferior, nasal, and temporal subfields, averaged amplitudes and implicit times from each subfield of the mfERG were also compared with the metamorphopsia scores at the corresponding subfields (Fig. 2).

### Statistical analysis

Comparisons of data between the ERM-affected eye and the control contralateral eye were performed using paired *t* test. In addition, one-way ANOVA (analysis of variance) with post *hoc* was performed for the subgroup analysis of the groups with no, mild, and severe metamorphopsia scores in the central subfield. A linear regression model was used to analyze the relationships among the degree of quantitative metamorphopsia, mfERG results, and OCT findings. Statistical analysis was performed using SPSS software (IBM SPSS Statistics for Windows, Version 21.0, IBM Corp., Armonk, NY). A *p*-value of < 0.05 was considered statistically significant.

## Results

### Demographic characteristics

In total, 52 eyes of 52 patients were included in this study. Of the enrolled patients, 28 were female, and 24 were males. Ten patients had previously undergone cataract surgery. The mean age of all patients was 65.3 ± 18.5 years (mean ± SD, range: 47–83 years). The mean initial BCVA of the ERM-affected eye was 0.50 ± 0.12, and that of the fellow eyes was 0.82 ± 0.26 (Table 1). Eleven patients had a history of hypertension, and five patients had a history of diabetes without diabetic retinopathy findings. Ten patients had previously undergone cataract extraction surgery.

**Table 1:** Demographic characteristics of enrolled idiopathic epiretinal membrane (ERM) patients

No. of patients (eyes)	52 (52)
Female:male	28: 24
Mean age (years)*	65.3 ± 18.5 (47–83)
Past history	
Hypertension	11
Diabetes mellitus	5
Pseudophakic eyes	10

^*^Mean ± standard deviation

### OCT findings

In OCT scans for the affected eyes, the retinal thickness for the central, superior, inferior, nasal, and temporal subfields on the ETDRS grid were 495 ± 102 µm, 428 ± 98 µm, 454 ± 78 µm, 434 ± 83 µm, and 463 ± 95 µm, respectively, while the corresponding retinal thickness of the control eyes was 270 ± 93 µm, 324 ± 83 µm, 321 ± 82 µm, 318 ± 90 µm, and 332 ± 87 µm, respectively (Table 2). Thus, the retinal thickness in all subfields of the affected eyes were significantly greater than those in the fellow eyes (*p* < 0.001). Furthermore, the retinal wrinkling ratios were 1.36 ± 0.11 in the ERM-affected eyes and 1.26 ± 0.19 in contralateral eyes. The difference in the retinal wrinkling ratios for both eyes was also statistically significant (*p* = 0.008) (Table 2).

**Table 2:** Baseline clinical characteristics in the ERM-affected eyes and contralateral eyes of the patients

	Affected eye	Contralateral eye	*p*-value^†^
BCVA (LogMAR)*	0.50 ± 0.12	0.82 ± 0.26	< 0.001
Retinal thickness on subfields (μm)			
Central	495 ± 102	270 ± 93	< 0.001
Superior	428 ± 98	324 ± 86	< 0.001
Inferior	454 ± 78	321 ± 82	< 0.001
Nasal	434 ± 83	318 ± 90	< 0.001
Temporal	463 ± 95	332 ± 87	< 0.001
Retinal wrinkling ratio	1.36 ± 0.11	1.26 ± 0.19	0.008

^*^Best-corrected visual acuity

^†^Analyzed using paired *t*-test

### Metamorphopsia scores

Metamorphopsia scores were obtained for a total of 21 subfields. The mean total metamorphopsia score across all subfields was 42.69 ± 24.93, while the corresponding mean total scores for the superior, inferior, nasal, and temporal subfields were 17.26 ± 9.39, 15.78 ± 9.75, 15.36 ± 9.24 and 16.63 ± 9.37, respectively. In addition, the scores for the central subfield was 2.12 ± 1.80 points. The mean individual subfield metamorphopsia score was 2.03 ± 1.18 (Table 3).

**Table 3:** Results of the metamorphopsia test in the affected eyes of the patients

	No. of subfields	Summation of metamorphopsia scores*	Mean metamorphopsia scores/ subfield*
Total field	21	42.69 ± 24.93	2.03 ± 1.18
Subfields			
Central	1	2.12 ± 1.80	2.12 ± 1.80
Superior	8	17.26 ± 9.39	2.07 ± 1.17
Inferior	8	15.78 ± 9.75	1.70 ± 1.22
Nasal	8	15.36 ± 9.24	1.92 ± 1.21
Temporal	8	16.63 ± 9.37	2.16 ± 1.17

^*^Mean ± standard deviation

**Fig. 3:**
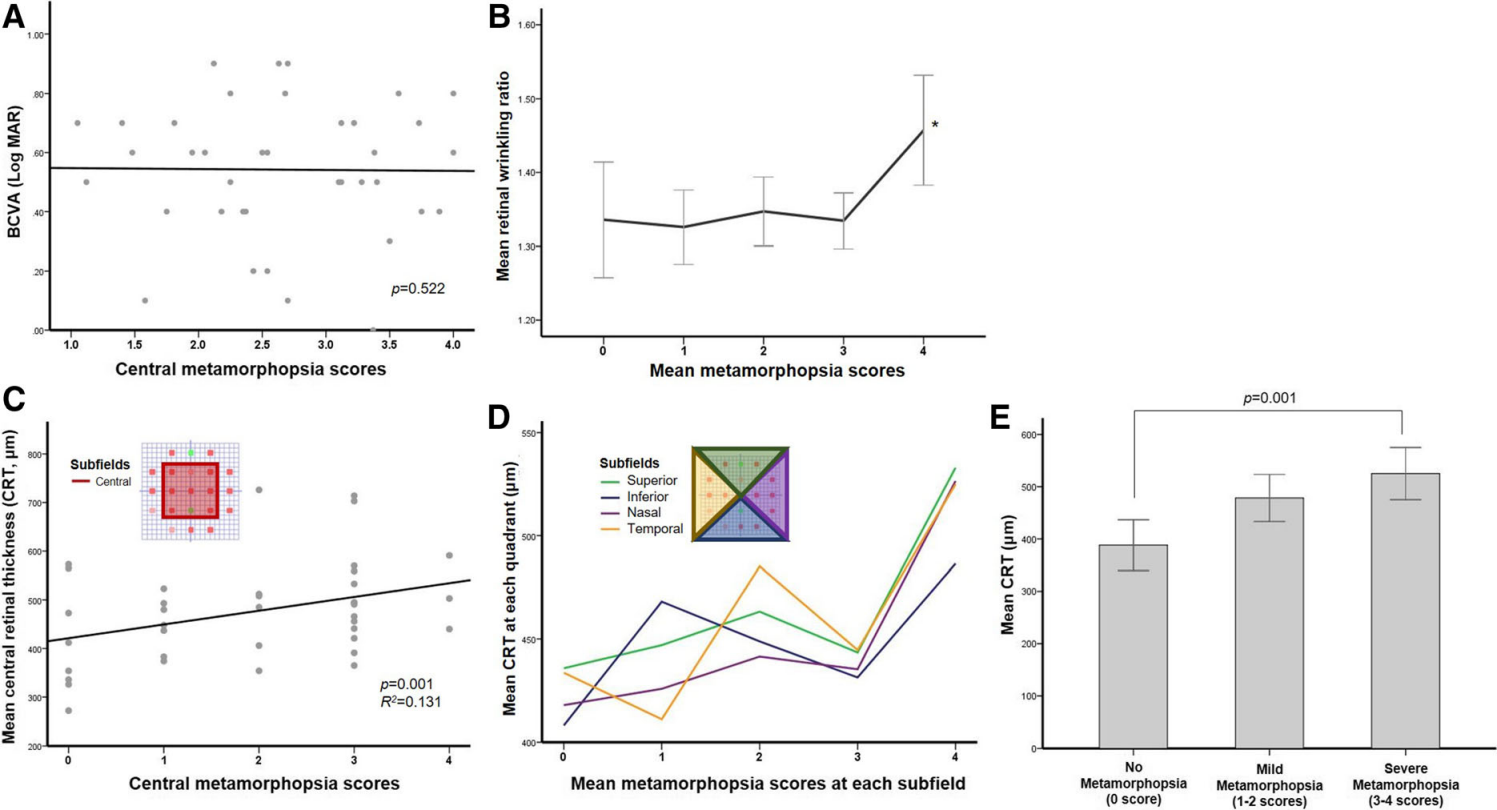
The correlations among metamorphopsia scores, best-corrected visual acuity (BCVA), and retinal thickness on OCT. There was no significant correlation between metamorphopsia scores and BCVA (*p* = 0.522) (**A**). The only subfield with a metamorphopsia score of 4 points had a significantly high retinal wrinkling ratio (*p* < 0.001) (**B** asterisk). The metamorphopsia scores for the central subfields presented significant correlations with central retinal thickness (CRT) (*p* = 0.001, *R*^2^ = 0.131) (**C**). The correlations between mean metamorphopsia scores and CRT for the individual subfields were complicated (**D**). Therefore, the patients were divided into subgroups as the no-metamorphopsia group (score, 0), mild-metamorphopsia group (scores, 1–2), and severe-metamorphopsia group (scores, 3–4). The mean CRT in the no metamorphopsia group was significantly different from that in the severe group (*p* = 0.001) (**E**). There was no significant difference in the CRT between the no- and mild-metamorphopsia groups, or between the mild- and severe-metamorphopsia groups

There was no significant correlation between metamorphopsia scores and BCVA (*p* = 0.522) (Fig. 3a). Moreover, metamorphopsia severities did not show a stepwise correlation with increases in the mean retinal wrinkling ratio under OCT scans. Only the subfield with a metamorphopsia score of 4 points had a significantly high retinal wrinkling ratio, compared to the other subfields with metamorphopsia scores from 0 to 3 points (*p* < 0.001) (Fig. 3b).

The metamorphopsia scores from the central subfields showed a significant correlation with CRT (*p* = 0.001, *R*^2^ = 0.131) (Fig. 3c). However, the correlations between the mean metamorphopsia scores and CRT according to the individual subfields were rather complicated (Fig. 3d). Therefore, the patients were divided into subgroups based on the severity of metamorphopsia in the central subfield, and in the no-metamorphopsia group (score, 0), mild-metamorphopsia group (scores, 1–2), and severe-metamorphopsia group (scores, 3–4). The mean CRT gradually increased, and the CRT in the no metamorphopsia group was significantly different from that in the severe metamorphopsia group (*p* = 0.001) (Fig. 3e). Unfortunately, there was no significant difference in the CRT between the no and mild metamorphopsia groups or between the mild and severe metamorphopsia groups.

### Correlations with mfERG parameters

**Fig. 4:**
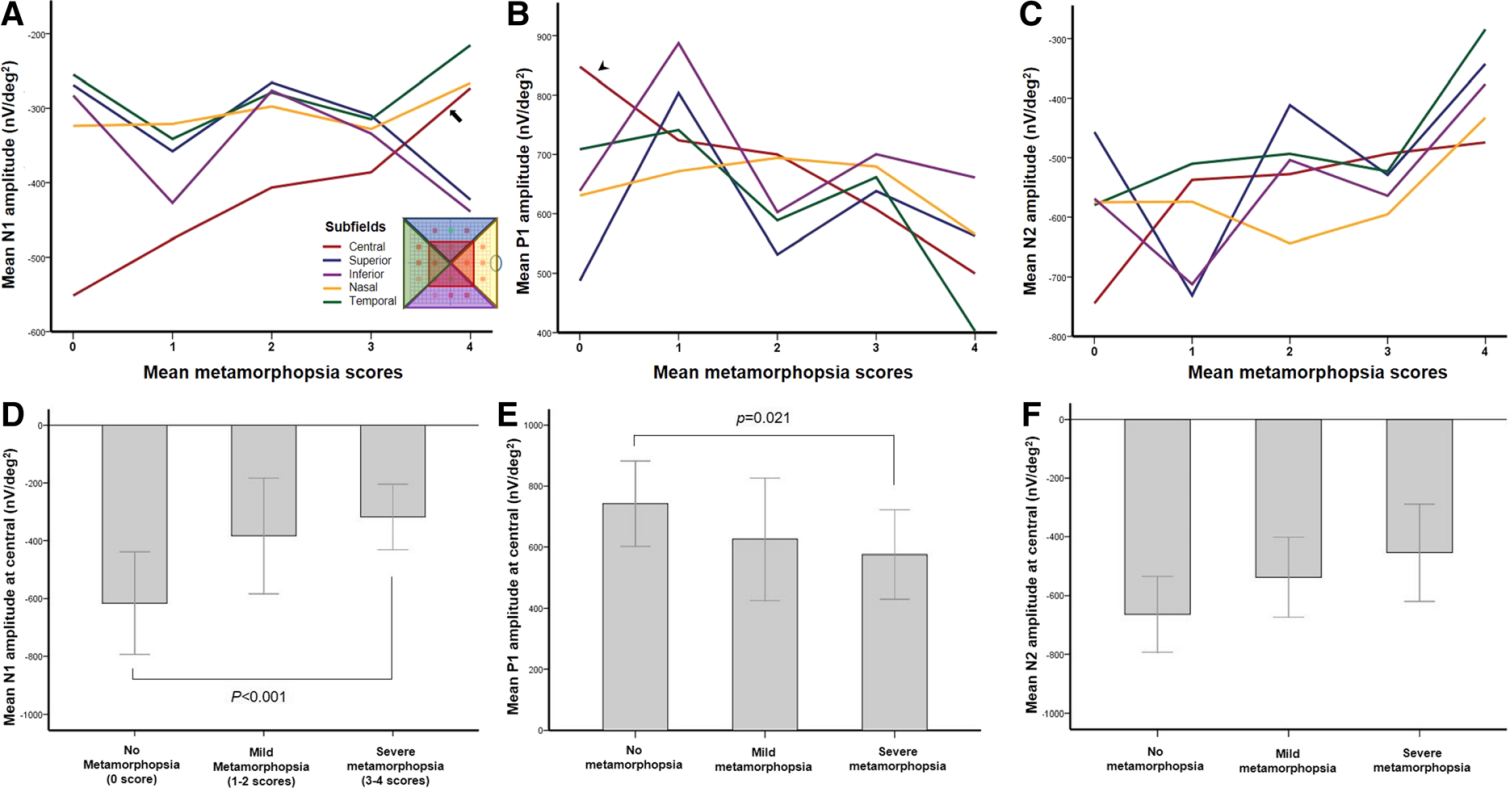
The correlations between metamorphopsia scores and mfERG amplitudes. The mean metamorphopsia scores in the central subfield showed a significant relationship with the mean N1 amplitude (*p* = 0.001, *R*^2^ = 0.081) (**A**, arrow), while other subfields showed no relationship between metamorphopsia scores and the N1 amplitudes. A significant correlation of metamorphopsia scores with the mean P1 amplitude was observed only in the central subfield (*p* = 0.048, *R*^2^ = 0.104) (**B**, arrowhead), not in the other subfields. However, metamorphopsia scores were not correlated with N2 amplitudes in all subfields (**C**). The mean N1 and P1 amplitudes in the severe metamorphopsia group was significantly lower than those in the no-metamorphopsia group (*p* = 0.001, *p* = 0.021, respectively) (**D** **E**), but not significant in the mild-metamorphopsia group. On the other hand, the mean N2 amplitude in mfERG showed no significant difference related to metamorphopsia severity (**F**)

The mfERG results for the ERM-affected eyes of the enrolled patients are shown in Table 4. The mean N1 amplitude of the central subfield was -439.8 ± 284.7 nV/deg^2^. The mean amplitudes of the P1 and N2 responses were 699.6 ± 378.6 and -585.9 ± 351.2 nV/deg^2^, respectively. The mean metamorphopsia scores in the central subfield showed a significant relationship with the mean N1 amplitude (*p* = 0.001, *R*^2^ = 0.081) (Fig. 4a), while the other subfields showed no relationship between the metamorphopsia score and N1 amplitude. Moreover, a significant correlation of the metamorphopsia score with the mean P1 amplitude was observed only in the central subfield (*p* = 0.048, *R*^2^ = 0.104) (Fig. 4b). However, the metamorphopsia scores were not correlated with the N2 amplitudes in all subfields (Fig. 4c).

**Table 4:** Results of multifocal electroretinogram (mfERG) in the affected eyes of the patients

	N1 amplitudes (nV/deg^2^)*	P1 amplitudes (nV/deg^2^)*	N2 amplitudes (nV/deg^2^)*
Subfields			
Central	−439.8 ± 284.7	699.6 ± 378.6	−585.9 ± 351.2
Superior	−318.1 ± 144.5	646.3 ± 263.1	−546.9 ± 273.7
Inferior	−331.4 ± 132.6	669.5 ± 217.1	−572.3 ± 198.6
Nasal	−316.7 ± 106.1	666.7 ± 197.8	−587.3 ± 188.1
Temporal	−305.4 ± 120.1	6643. ± 232.8	−509.0 ± 243.5

	N1 implicit times (ms)*	P1 implicit times (ms)*	N2 implicit times (ms)*
Subfields			
Central	28.7 ± 3.5	47.5 ± 4.4	66.9 ± 8.2
Superior	29.6 ± 4.0	48.8 ± 5.1	67.8 ± 9.2
Inferior	28.9 ± 3.8	48.4 ± 4.7	68.1 ± 6.7
Nasal	29.2 ± 3.1	48.2 ± 4.9	67.6 ± 8.7
Temporal	29.5 ± 4.2	47.9 ± 3.8	68.0 ± 7.5

^*^Mean ± standard deviation

Similar to the analysis using CRT, the correlations of metamorphopsia scores with the averaged N1, P1, and N2 amplitudes were also analyzed after dividing the patients into subgroups based on metamorphopsia severity (Fig. 4). The mean N1 and P1 amplitudes gradually decreased according to metamorphopsia severity (Fig 4d–f). The mean N1 amplitude of the severe metamorphopsia group was significantly lower than that of the no metamorphopsia group (*p* = 0.001) (Fig. 4d) and did not significantly differ from that of the mild-metamorphopsia group. The mean P1 amplitude of the severe metamorphopsia group was also significantly lower than that of the no metamorphopsia group (*p* = 0.021) (Fig. 4e). Conversely, the mean N2 amplitude in mfERG showed no significant difference related to metamorphopsia severity (Fig. 4f).

**Fig. 5:**
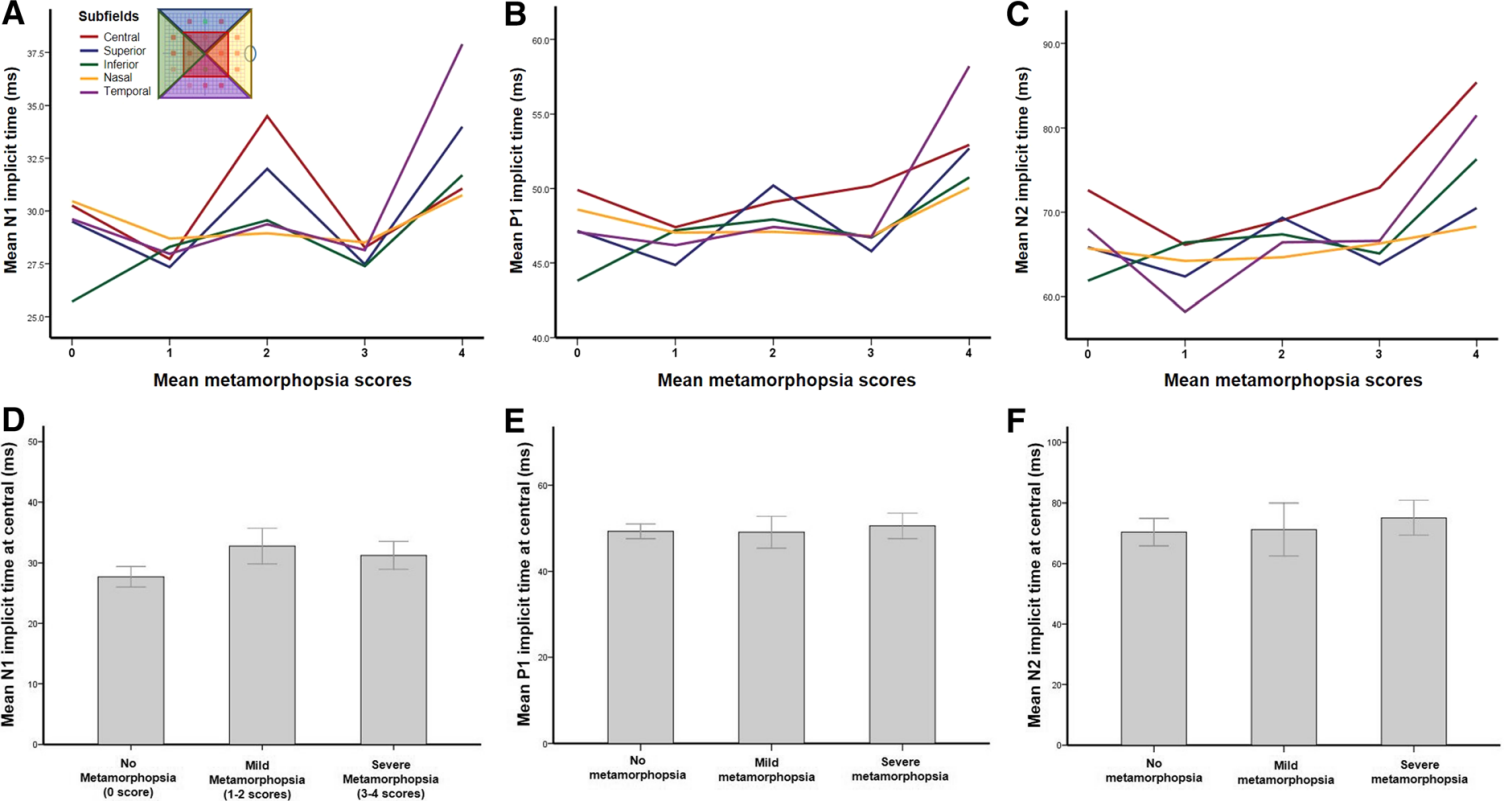
The correlations between metamorphopsia scores and mfERG implicit times. Unlike the amplitudes, implicit times in mfERG did not show significant change related to metamorphopsia scores at all subfields (**A**–**C**). There was also no significant difference in the N1, P1, and N2 implicit times according to metamorphopsia severity (**D**–**F**)

Unlike the results for amplitudes, implicit times from mfERG did not show significant changes with metamorphopsia scores at all subfields (Fig. 5). There were also no significant differences in the N1, P1, and N2 implicit times in the subgroups categorized by metamorphopsia severity (Fig. 5).

## Discussion

Metamorphopsia is a common symptom presenting in about 85% of patients with ERM, and this condition has been evaluated using the Amsler grid or M-chart [2, 5, 12]. The Amsler grid has been primarily used for assessing and quantifying metamorphopsia in macular diseases. However, this tool is inaccurate and cannot easily quantify the severity of metamorphopsia. Bouwens and Van Meurs attempted to quantify metamorphopsia using the Sine Amsler grid, but they found that it was not suitable for general clinical situations and was limited with regard to objective quantification [2]. Lakshminarayanan et al. and Shinoda et al. also attempted to quantify metamorphopsia by modifying the Amsler grid, but their method could not be used clinically because of its complexity [4, 13]. On the other hand, assessments based on the M-chart can quantify metamorphopsia more objectively than those with the Amsler grid by determining only the deflection of the line seen by the patient. However, this approach also has the disadvantage that it is difficult to determine the degree of metamorphopsia part by simply showing only one horizontal or vertical line at the center of the field [5].

As described above, quantification of metamorphopsia in patients with ERM has been attempted in various previous studies, but there is no clearly established objective evaluation method. To address these issues, a novel objective metamorphopsia test was proposed, that uses assessments similar to those of automatic visual field perimetry. The reproducibility of this metamorphopsia test has already been shown in our previous domestic paper [14]. In this study, therefore, the relationship between retinal structure and quantitative metamorphopsia could be clarified, and the correlation between the localized retinal function and the degree of metamorphopsia in the area corresponding to the subfields was also identified using OCT scans and mfERG findings.

Visual acuity was not correlated with metamorphopsia scores in this study. Visual acuity was relatively maintained despite an increase in the metamorphopsia scores. This suggests that visual disturbances and metamorphopsia are separate symptoms and that metamorphopsia does not just necessarily lead to visual impairment in patients with ERM.

The metamorphopsia scores were correlated with the retinal thickness measurements obtained with OCT scans in this study. Several previous studies have evaluated the correlations between metamorphopsia symptoms and retinal structural findings detected using OCT. Metamorphopsia induced by ERM was related to the edematous areas of the retina measured using OCT [15]. In particular, the inner nuclear layer of the retina was a potentially useful indicator for ERM surgery. Recently, ectopic inner foveal layers based on the OCT-based grading scheme in patients with advanced stages of ERM were considered a good indicator for metamorphopsia [16]. A previous paper reported that the degree of metamorphopsia was also reduced after successful ERM removal surgery [17]. However, in most studies, the M-chart was used to quantify the metamorphopsia symptoms of the patients with ERM. In this study, a more objective metamorphopsia testing method was used, and we tried to investigate the correlation of the degree of metamorphopsia with retinal structural characteristics, including retinal thickness and retinal wrinkling. The results showed that the metamorphopsia degree was significantly correlated with retinal thickness only in the central retina. Unfortunately, this relationship was not identified in the subfields, other than the central subfield. The retinal wrinkling ratio also only showed a correlation in the patient group with high metamorphopsia scores.

Many previous studies have attempted to evaluate the retinal function in patients with ERM using mfERG recordings and OCT scans. Photoreceptor disruption found on OCT scans and the delay of the P1 implicit time on mfERG were significant predictors of visual prognosis after ERM removal surgery [10]. CRT and inner retinal layer thickness obtained with OCT were strongly correlated with BCVA and the P1 amplitude of mfERG [11]. The retinal functional status in patients with ERM was evaluated using mfERG, and the correlations of the degree of metamorphopsia with retinal structural findings at each subfield were also investigated. There was a significant correlation between the mfERG amplitudes and metamorphopsia scores. Similar to the results between metamorphopsia degree and retinal thickness, the degree of metamorphopsia showed a significant relationship with the N1 and P1 amplitudes in the central subfield. We attempted to determine if there was a significant correlation between the metamorphopsia scores and mfERG responses in the superior, inferior, nasal, and temporal subfields, but we could not find a definite correlation. After dividing the patients into subgroups based on metamorphopsia severity, the N1 and P1 amplitudes were different between the no- and severe-metamorphopsia groups; however, a stepwise significant relationship was not identified. Moreover, the implicit times of mfERG did not change according to the severity of metamorphopsia symptoms. Taken together, several correlations among metamorphopsia, retinal structural integrity assessed with OCT, and localized retinal function elicited from mfERG were found, especially for the central retina. The retinal areas with a metamorphopsia score of 4 points showed greater retinal thickness, a more wrinkled retinal structure, and reduced N1 and P1 amplitudes in the mfERG response.

However, the correlations were observed mostly in the corresponding area of central retina. The outermost line of each grid in the metamorphopsia test represents a range of 10°. The central retinal thickness using the ETDRS grid in the OCT scan measures the average thickness of the retina with a diameter of 1 mm. In mfERG recording, Ring 1 and Ring 2 represented a field of 5°. It is not rigorous enough to perform correlation analysis with the measurements carried out at different extension. It is suggested that correlations were rarely observed outside the central retina, because of this inconsistency of the extension among the examinations.

This study had several limitations. First, this study was limited by the small size of the study population. The present results should be validated with a large sample. Second, because this study was a cross-sectional observational study, we could not assess the consecutive changes in metamorphopsia and mfERG findings during the natural course of the disease. In the future, alterations in metamorphopsia score and mfERG results should be evaluated for longer periods and compared with the results obtained after ERM removal surgery. If the postoperative changes in metamorphopsia scores are investigated, these scores may be used as an essential criterion or indicator to decide regarding surgical treatment in patients with ERM. Third, the proficiency in performing the metamorphopsia test may have affected the results, similar to an automated visual field perimetry. The metamorphopsia test is a new method, and several repeated measurements may be required for each patient. Fourth, it is possible that the subfields of the metamorphopsia test, the ETDRS grid of OCT, and the concentric ring of mfERG do not exactly match each other, as discussed in the previous paragraph. The test field on the retina was divided into central, superior, inferior, nasal, and temporal subfields in this study; accordingly, some subfields might have ambiguous boundaries while others might have overlapped. Fifth, different types or degrees of cataract in the patients may influence the symptoms of metamorphopsia, although the patients with lens opacity of grade 2 or higher in the LOCS system were excluded from this study. Clinical data associated with the lens state of the patients with ERM were overlooked. Nevertheless, this study is meaningful in that the degree of metamorphopsia symptoms in patients with ERM was compared with the retinal structure and retinal function at localized subfields.

In conclusion, the degree of metamorphopsia for each subfield was objectively quantified using the metamorphopsia test, and metamorphopsia scores in various subfields of patients with ERM were obtained. The degree of metamorphopsia was correlated with central retinal thickening on OCT. mfERG also demonstrated significant reduction of localized retinal function in the central subfields, and this reduction correlated with the metamorphopsia scores. The metamorphopsia test can be a useful method to evaluate patients with ERM complaining of metamorphopsia symptoms. These results can be applied to other macular diseases, such as macular hole or age-related macular degeneration, and they may improve our understanding of the changes in metamorphopsia following treatments, including vitreous retinal surgery.
